# An evaluation method for product design solutions for healthy aging companionship

**DOI:** 10.3389/fpubh.2022.919300

**Published:** 2022-09-20

**Authors:** Shan Hu, Qi Jia, Linlin Dong, Jialin Han, Min Guo, Weiqi Guo

**Affiliations:** School of Industrial Design, Hubei University of Technology, Wuhan, China

**Keywords:** healthy aging, product solutions decision, comprehensive evaluation method, game theory portfolio empowerment, rooted in theory, TOPSIS method

## Abstract

**Background:**

With the development trend of healthy aging and intelligent integration, escort products have become a new means of healthy aging. Healthy old-age care pays attention to the convenience and informatization of life. To meet the needs, designers often design multiple accompanying product solutions, and it is very important to use reasonable evaluation methods to decide on the optimal solution.

**Purposes:**

A new comprehensive evaluation method is proposed to reduce the subjectivity and one-sidedness of the selection process of intelligent escort product design solutions, and to make the decision more objective and reasonable. Such decisions can enhance the experience and naturalness of the elderly using intelligent products.

**Methods:**

First, a large number of user interviews were analyzed using the grounded theory, gradually refine through theoretical coding, and abstracted with the design scheme evaluation index. Second, the idea of game-theoretic weighting is used to optimize a linear combination of subjective and objective weights to determine the final weights of each evaluation indicator. Finally, the evaluation and selection are completed based on the solution ranking determined by the approximate ideal solution ranking method (TOPSIS). It is applied for the selection of the elderly escort robot design, and the usability test is conducted using the PSSUQ to verify the selection results.

**Results:**

A new comprehensive evaluation method can better complete the preferential selection of product design solutions for healthy aging escorts, and reduce the subjectivity and one-sidedness of the evaluation.

**Conclusion:**

This method compensates for the reliance on personal experience in the selection of options, and improve the subjectivity of the evaluation index determination process and the deviation of index weighting. Improving the objectivity and scientificity of decision-making reduces the blindness of design and production. It also provides a theoretical reference for the research scholars of healthy aging companion products.

## Introduction

With the demographic changes, Chinese society has shifted from mild aging to moderate aging, and the accompanying health care has become the focus of social attention. Relying on the maturity and popularity of information technology, smart aging as a new concept began to be widely used in the field of elderly care. Its purpose is to use smart products to help the elderly improve the convenience and informatization of their lives and achieve healthy retirement. For example, (1) summarized and elaborated on the introduction and development of the concept of smart wellness, which can enhance the possibility of self-care for the elderly and reduce the burden of elderly care through the popular application of smart platforms and products. Anghel et al. (2) investigated that intelligent environments and intelligent technologies, such as machine learning and robot assistance, can support the independent living of the elderly and provide friendly nursing services to enable the elderly to have a safer and more independent healthy aging. At the same time, there are more and more researches on smart products for the elderly, from the aspects of product user needs, product solution conception, product development, usability testing, and user acceptance. For example, Neira-Rodado et al. (3) explained the changing needs, ambiguous user requirements, and interrelationships between design criteria; Propose a new intelligent product design process that directly translates user needs into product functions, and validates the new intelligent product design process with hip replacement surgery for the elderly. Wei et al. (4) conducted a research on smart wearable health monitoring products for preventing elderly diabetes, including user information source channels, concerns, and satisfaction with the product, and verified the proposed scheme through a large number of experimental evaluations to solve the problem **(**said problem). Daher et al. (5) developed an indoor tracking and fall detection system for the elderly, based on smart tiles to detect falls of the elderly, locate and identify human activities, and provide necessary assistance when needed. Kim et al. (6) developed usability testing standards for elderly communication service robots from four aspects: safety, controllability, efficiency, and satisfaction, which are a good measurement tools to help users and developers of elderly service robots. Ghorayeb et al. (7) discussed older adults' perceptions of smart home technology and developed sensor platforms for home health and lifestyle and found that older adults' acceptance increases with time and use. In addition, it is also very important to select smart products that meet the needs of the elderly from the design scheme.

In the design stage of information technology products, designers cannot objectively and rationally select the one that best meets the needs of users among various design schemes. As a result, the final product does not conform to the cognitive behavioral habits of the elderly and reduces the quality of healthy life of the elderly, so healthy aging cannot be achieved. At the same time, healthy aging is reflected in all aspects of daily life. Through design, the needs of the elderly are solved one after another, not only to meet the needs of the elderly but also to meet personal expectations through the optimization of design solutions. The optimal decision of product design scheme is an important link in the overall design process which can reduce design blindness, and improve objectivity and scientificity (8). The evaluation results can affect the value and performance of the product and even the production cost of the company (9), which ultimately and indirectly affects elderly users and their daily lives. The optimization of the design scheme involves multiple criteria and multiple objectives. In the actual design decision-making process, designers often evaluate and select programs based on personal experience, lack of uniform standards, at the same time, as a special group of the elderly, their cognitive ability continues to decline, and smart products should be designed to reduce their cognitive difficulties. Therefore, many scholars choose the method according to the existing design scheme, and draw on the knowledge research and evaluation methods of operations research, management, mathematics and other disciplines, mainly including AHP (10–12), entropy weight method (13–15), TOPSIS Method (16–18), and comprehensive evaluation method (19–21), etc. Continuously experiment and summarize the optimal method of design scheme. In the literature mentioned above, many scholars often use expert interviews and questionnaires to establish evaluation indicators and evaluation systems, and the hierarchy of interviewees is randomly selected during the interviews. At the end of the interviews, the raw material obtained was not refined by scientific theories and analytical tools, but rather consolidated on the basis of subjective perceptions. Indicators are often derived based on direct induction by the researcher, which is highly subjective, unsupported by theory, and one-sided; and the establishment of evaluation indicators lacks guidance from qualitative research methods. The weight value calculation process is often a single calculation method, and even the combined weighting method uses the conventional multiplicative normalization (22) and linear weighting of subjective preference coefficients when combining subjective and objective weights, so that the multiplicative effect problem of larger and smaller weight values will exist subjectively and arbitrarily, which eventually causes bias for the evaluation results.

Faced with the shortcomings of the existing evaluation methods in the optimization of the scheme, grounded theory and game theory combined empowerment are introduced. Grounded theory is a bottom-up qualitative theoretical research method and is one of the scientific qualitative research methods (23). The researcher does not make theoretical assumptions at the beginning of the study, but starts directly from practical observations and finds the core concept representing the essence of things from the systematically collected data, which is characterized not by its empirical nature, but by the new concepts and ideas it abstracts from the empirical facts. More and more scholars are applying grounded theory to qualitative research in various fields. For example, Goodall et al. (24) used grounded theory to study how older people who “age in place” find health information and the role that digital technology plays in this. Seesawang et al. (25) used grounded theory to develop a theory on the perceived risk experience of older men with hypertension in rural Thailand. Wang et al. (26) applied it to the study of factors influencing the demand for smart health care. In addition, the grounded theory uses a semi-structured interview method, which is somewhat free and open, is not influenced by existing views, is able to discover factors that are overlooked in current ideas or theories, and to a certain extent can scientificize and objectify inductive summaries that rely on experience. At the same time, many scholars have proposed the use of two or more evaluation methods to combine the weighting, to avoid a single method to determine the weight of indicators is too one-sided and no reference value; game theory combination of weighting is one of them, which is widely used by many scholars for comprehensive evaluation. For example, Men et al. (27) used it for the evaluation decision of power equipment suppliers to provide a reference. Geng and Bo (28) used it to analyze the relationship between customer needs. Game theory combination assignment adopts the idea of reducing the deviation of subjective and objective weights to fully combine the advantages of the two, to a certain extent to improve scientific rationality, not only to avoid the problem of subjective arbitrariness in the subjective assignment, but also to avoid the problem of objective assignment based only on inherent information without highlighting the relative importance of indicators.

In summary, we found that many scholars focus on product development, mostly the idea research before product development and the method research of the development process. Few scholars pay attention to the evaluation stage of the product scheme, while the evaluation of the product design scheme is related to scheme optimization, value maximization, inaccurate evaluation and lack of screening of solutions will lead to blindness in production, affecting product performance or even not meeting user needs. In addition, designers do not have an objective evaluation decision model as a reference for program selection, which makes designers' decision-making vague and subjective, resulting in unsatisfactory results. Therefore, it is very necessary to establish an objective evaluation decision model and apply it to the selection of intelligent escort product solutions. The paper aims to apply the combination of grounded theory and game theory to improve the subjectivity of the index determination process and the bias of the index assignment, and then use the TOPSIS method to decide on the optimal solution, to propose a new comprehensive evaluation method combining qualitative and quantitative research methods, improve the objectivity and science of the evaluation. Providing theoretical references for enterprises and designers to help them evaluate the optimal solution to serve the elderly better and improve the user experience and the health of the elderly life.

## Method

### Theoretical overview

#### Grounded theory

Grounded Theory was proposed by sociologists Glaser and Strauss (29), and its main idea is to build a theory based on empirical information, summarize and gradually refine the information obtained from semi-structured interviews, and then rise to a systematic theory. On the basis of systematically collecting data, the core concepts that reflect the essence of things and phenomena are sought (30), and then relevant social theories are constructed through the connections between these concepts. The process is problem selection, data collection, data analysis, theory building, theory saturation testing, and conclusion formation. Open, spindle, and selective coding are the three key steps in forming a theory, and when theory saturation does not meet the criteria, then further data collection and analysis are required for validation.

#### Game theory portfolio empowerment

Game theoretic portfolio assignment is based on the idea of the game set model, which coordinates conflicts between different decision makers and seeks consistency, minimizing the deviation between each weight and the optimal weight, so as to obtain a relatively balanced and coordinated combined weight vector, and increases the accuracy of weights (31). The method can combine the advantages of subjective and objective assignments, find agreement and compromise between subjective and objective weights with Nash equilibrium as the coordination objective, and find the maximum common interest among indicators. It can take into account both subjective and objective weights, optimize their combination, and improve the scientific rationality of indicator assignments. It not only takes into account the inherent information of indicators but also reduces the subjective arbitrariness and avoids the weighting values from being too one-sided.

### Comprehensive evaluation method construction

**Figure 1:**
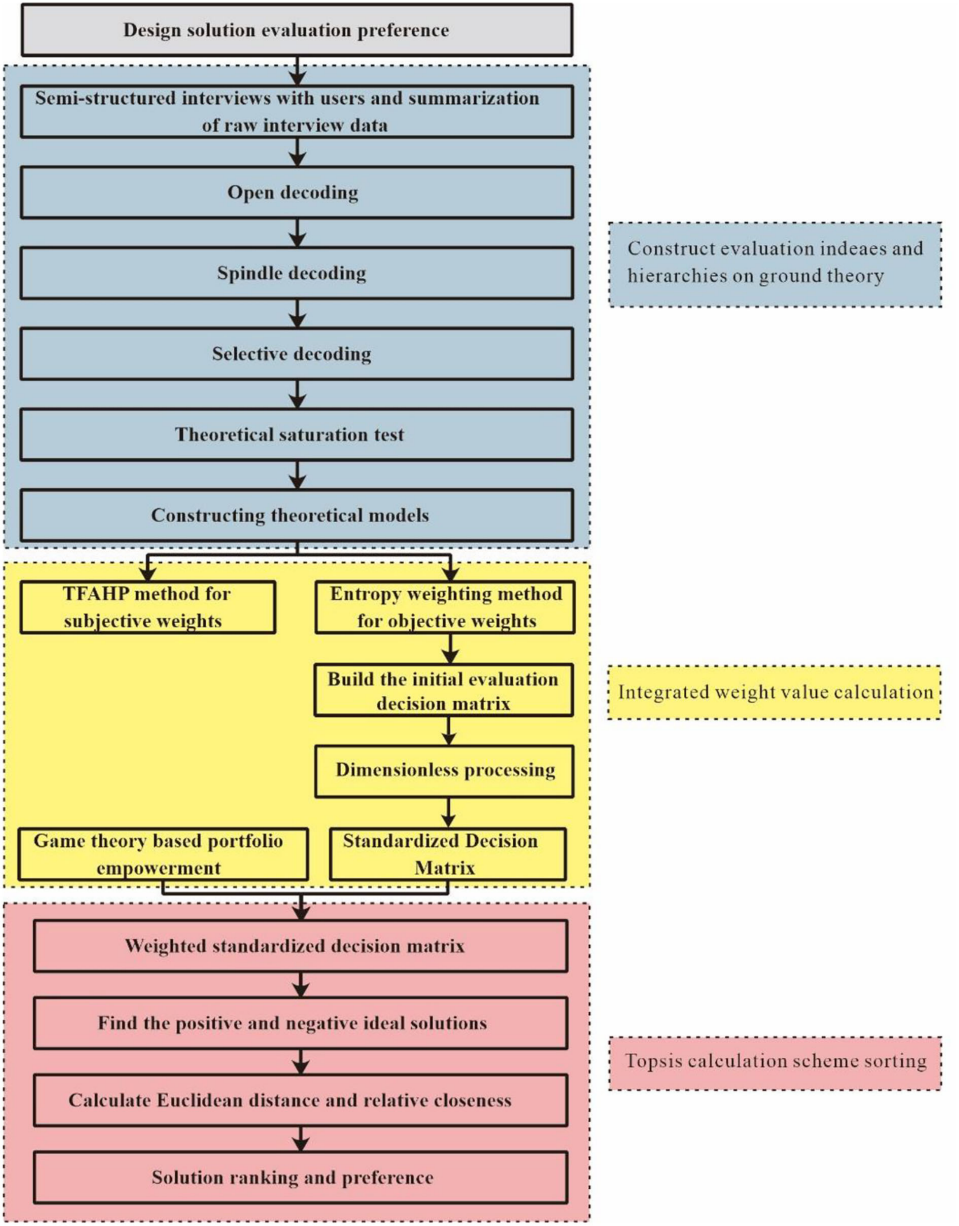
Comprehensive evaluation decision model.

The evaluation process of the new comprehensive evaluation method proposed in the paper is divided into three parts. In the first part, based on the grounded theory, the semi-structured interview data are summarized, analyzed, and coded step by step to extract the evaluation indicators, and the corresponding evaluation hierarchy system is constructed. In the second part, the subjective and objective weights of the evaluation indicators are calculated by the subjective weighted fuzzy analytic hierarchy process and the objective weighted entropy method. The linear combination of the two weights is optimized using the game theory combination weighting, and the combination weight is derived. In the third part, the combined weights of the evaluation indicators are applied to the ranking calculation of the TOPSIS scheme, and the preferred scheme is obtained. The evaluation decision model is constructed as shown in Figure 1.

#### Evaluation levels and indicator construction

The establishment of evaluation criteria and indicators is based on an in-depth analysis of the original information based on the grounded theory, refining and summarizing the hidden laws as well as connections, so as to form a theory that is the evaluation hierarchy and indicators. Therefore, the selection of interviewees should be representative and extensive, preferably with frequent contact with the evaluated subjects and a strong willingness to cooperate, to ensure the comprehensiveness of data collection.

#### Integrated weight value calculation

The triangular fuzzy analytic hierarchy process (TFAHP) is based on the analytic hierarchy process (AHP), which introduces a triangular fuzzy number set and transforms the fuzziness of the qualitative index evaluation process into corresponding fuzzy numbers for quantification. This not only retains the advantages of AHP but also avoids the inaccuracy of evaluation caused by the fuzziness of human thinking and the structural redundancy caused by the consistency test.

The basic idea of the entropy weight method is to determine the objective weight according to the size of the degree of difference of indicators. The degree of difference is the size of the entropy weight of the indicator, and the greater the entropy weight of the evaluation indicator, the greater the degree of contribution of the indicator to the product evaluation.

##### Subjective weighting calculation

First, the evaluation criteria were scored for two comparisons to construct a fuzzy mutual inverse judgment matrix for the target layer. Judges were asked to use a scale of 1–9 to judge the adjudication, and the quantification and meaning of the defined terms are shown in Schedule S1 Table. The general process of triangulated fuzzy hierarchical analysis is as follows (32, 33):

(1) Taking the target layer element Q and its corresponding first-level criterion *M*_1_, *M*_2_, …, *M*_*n*_ of the criterion layer as an example, the fuzzy mutual inverse judgment matrix is constructed as follows:
  (1) $$\begin{array}{l} \begin{array}{c} A={\left(r_{ij}\right)}_{n\times n} \end{array} \end{array}$$
  Where the fuzzy set of *r*_*ij*_ is (*l*_*ij*_, *m*_*ij*_, *u*_*ij*_), and the n-term criterion is compared for judgment, then we get:
  (2) $$\begin{array}{l} A=\left[\begin{array}{ccccc} r_{11} & r_{12}r_{13} & \cdots & r_{1n}\\ r_{21} & r_{22} & r_{23} & \cdots & r_{2n}\\ r_{31} & r_{32} & r_{33} & \cdots & r_{3n}\\ \vdots & \vdots & \vdots & \ddots & \vdots \\ r_{n1} & r_{n2} & r_{n3} & \cdots & r_{nn} \end{array}\right] \end{array}$$
(2) The criteria of each level of each scheme were scored by z experts, and the data were aggregated and processed using the Delphi method (34) to establish the *Q* − *M* level fuzzy mutual inverse judgment matrix. Assuming that the scorers have similar levels of knowledge as well as judgment, the arithmetic average method is used to synthesize the scoring information, and thus a comprehensive fuzzy mutual inverse judgment matrix for the *A* − *R* layer can be obtained as follows.
  (3) $$\begin{array}{l} \begin{array}{c} A={\left(\frac{\sum_{t=1}^{z}l_{ij}^{t}}{z},\frac{\sum_{t=1}^{z}m_{ij}^{t}}{z},\frac{\sum_{t=1}^{z}u_{ij}^{t}}{z}\right)}_{n\times n}\;\\ ={\left(L_{ij},M_{ij},U_{ij},\right)}_{n\times n} \end{array} \end{array}$$
  Where, *r*_*ij*_ denotes the importance between element *i* and element *j*, and $r_{ij}=r_{ij}^{-\;1}$.
(3) Defuzzification of the matrix: The mean area method is generally used, as shown in Equation (4).
  (4) $$\begin{array}{l} \begin{array}{c} A={\left(\frac{L_{ij}+2M_{ij}+U_{ij}}{4}\right)}_{n\times n}={\left(r_{ij}\right)}_{n\times n} \end{array} \end{array}$$
(4) Weight value calculation: After the defuzzification process, the calculation of the weight vector of each matrix is started, and since the calculation steps are common calculation methods, the specific procedure is referred to the literature (35).
(5) Consistency test: Due to the vagueness and limitations of human thinking and the complexity of judging things, there may be inconsistencies in the judgments given by decision-makers in the judging process, so the consistency of the matrix needs to be tested, and the selection of the average random consistency criterion *RI*, *CI*, and the calculation steps of the consistency ratio *CR* refer to the literature (36).

##### Objective weighting calculation

When determining the objective weights using the entropy weighting method, the entropy weighting coefficient method is used to calculate them. The general process is as follows (37):

(1) To express the degree of variation in the evaluation of evaluation indicators by different decision makers, the entropy of evaluation attributes is calculated using the following equation:
  (5) $$\begin{array}{l} \begin{array}{c} \;H_{j}=-k\overset{m}{\underset{i=1}{\sum }}p_{ij}\ln\left(p_{ij}\right)\\ =-\frac{1}{\ln\left(m\right)}\overset{m}{\underset{i=1}{\sum }}p_{ij}\ln\left(p_{ij}\right),\;j=1,2,\ldots ,n \end{array} \end{array}$$
  It is assumed that ln(*p*_*ij*_)=0 when *p*_*ij*_ = 0. The higher the value of 1 − *H*_*j*_, the more important the evaluation index is, and the higher the weight. The entropy weight of the evaluation index can then be expressed by the following equation:
  (6) $$\begin{array}{l} w_{j}=\frac{1-H_{j}}{n-\sum_{j=1}^{n}H_{j}},\;j=1,2,\ldots ,n \end{array}$$
(2) Construct a comprehensive fuzzy decision matrix. From the z scorers in the previous TFAHP using the linguistic variables fuzzy numbers in Schedule S2 Table to evaluate the *n* indicators of *m* sets of solutions, a fuzzy initial decision matrix is constructed, which is also expressed by triangular fuzzy numbers, denoted as *g*_*ijz*_ = {(*l*_*ijz*_, *m*_*ijz*_, *u*_*ijz*_)|*i* = 1, 2, …*m, j* = 1, 2, …*n, z* = 1, 2, … *Z*, }. After aggregating the evaluation values of multiple evaluators, each element of the decision matrix *g*_*ij*_ = {(*l*_*ij*_, *m*_*ij*_, *u*_*ij*_)|*i* = 1, 2, …*m, j* = 1, 2, …*n* }:
  (7) $$\begin{array}{l} \{\begin{array}{c} l_{ij}=\left\{l_{ijz}\right\}\\ mij=\frac{1}{Z}\sum_{z=1}^{Z}\left\{m_{ijz}\right\}\\ u_{ij}=\left\{u_{ijz}\right\} \end{array} \end{array}$$
  The mean area method is used to defuzzify the process. The equation is as follows:
  (8) $$\begin{array}{l} F={\left[f_{ij}\right]}_{m\times n},f_{ij}=\frac{\left(l_{ij}+2m_{ij}+u_{ij}\right)}{4} \end{array}$$
  Normalization of the matrix:
  (9) $$\begin{array}{l} p_{ij}=\frac{f_{ij}}{\sum f_{ij}},\;i=1,2,\ldots ,m \end{array}$$
(3) The objective weights were calculated from the entropy weight coefficient method using Equations (5) and (6).

##### Game theory portfolio empowerment

The game theory-based combined assignment method is to minimize the deviation of subjective and objective weights to find the maximum common interest among indicators. The steps are as follows (38):

(1) Basic weight vector set *w*_*k*_ = {*w*_*k*1_, *w*_*k*2_, …, *w*_*kn*_}(*k* = 1, 2, …, *K*), *n* is the number of evaluation indicators of the evaluated object, *K* is the number of weighting methods, and *K* is taken as 2. Let the linear combination of weighting coefficients α = {α_1_, α_2_, …, α_*K*_}. Any linear combination of these vectors is:
  (10) $$\begin{array}{l} w=\overset{n}{\underset{k=1}{\sum }}\alpha_{k}w_{k}^{T} \end{array}$$
  Where: *w* is a linear combination of weights; α_*k*_ is the weight coefficient; $w_{k}^{T}$ is the transpose matrix of the basic weight vector set *w*.
(2) Optimal combination: *K* linear weight combinations with coefficients α_*K*_ are optimized to obtain the most satisfactory weights. Then the objective function is:
  (11) $$\begin{array}{l} \underset{k}{\min}\left\|\overset{n}{\underset{k=1}{\sum }}\alpha_{k}w_{k}^{T}-w_{k}\right\|2\left(k=1,2,\ldots ,K\right) \end{array}$$
  Where, α_*k*_ is the weight coefficient; $w_{k}^{T}$ is the transpose matrix of the basic weight vector set *w*_*k*_; *w*_*k*_ is the basic weight vector set.
(3) Combination weights: After normalizing the obtained optimized combination coefficients α_*k*_, the combination weights $w^{*}=\left(w_{1},w_{1},\ldots ,w_{n}\right)$ are obtained by the following equation:
  (12) $$\begin{array}{l} w^{*}=\overset{n}{\underset{k=1}{\sum }}\alpha_{k}*w_{k}^{T} \end{array}$$
  where, w^*^ is the weight of the game theory portfolio assignment; $\alpha_{k}*$ is the weight coefficient after normalization process.

#### Scheme sorting and selection

There are many methods for multi-attribute decision problems (39, 40), and the TOPSIS method is the more commonly used, it is an intuitive and easy-to-understand method. This method can use the distance between each scheme and the positive and negative ideal solutions in the multi-dimensional space to measure the pros and cons of the scheme. The principle is derived from the theory of compromise planning and reference solution, which is in line with the logic and habit of comparing with a certain index as a reference in the decision-making of product design scheme optimization. The initial decision matrix of the entropy weighting method in which *z* scorers evaluate *n* indicators of *m* sets of solutions is dimensionless according to Equation (13) to construct the standardized decision matrix *X* = (_*x*_*ij*_)*m*×*n*_.


(13) $$\begin{array}{l} x_{ij}=\{\begin{array}{c} \left(\frac{l_{ij}}{u_{j}^{+}},\frac{m_{ij}}{m_{j}^{+}},\frac{u_{ij}}{l_{j}^{+}}\wedge 1\right),The\;bigger\;the\;better\;type;\\ \;\left(\frac{l_{j}^{-}}{u_{ij}},\frac{m_{j}^{-}}{m_{ij}},\frac{u_{j}^{-}}{u_{ij}}\wedge 1\right),The\;smaller\;the\;better\;type. \end{array} \end{array}$$

Multiplying this with the combination weights yields the weighted normalized decision matrix *Y* = (*y*_*ij*_)_*m*×*n*_ as:


(14) $$\begin{array}{l} y_{ij}=w_{j}p_{ij}\left(i=1,2,\ldots ,m;j=1,2,\ldots ,n\right) \end{array}$$

In the constructed weighted normalized decision matrix *Y*, the positive ideal solution *Y*^+^, the optimal vector consisting of the maximum value of each sub-criterion, and the negative ideal solution *Y*^−^, the worst vector consisting of the minimum value of each sub-criterion is identified.


$$\begin{array}{l} Y^{+}=\left\{Y_{1}^{+},Y_{2}^{+},\ldots ,Y_{m}^{+}\right\}\\ Y^{-}=\left\{Y_{1}^{-},Y_{2}^{-},\ldots ,Y_{m}^{-}\right\} \end{array}$$

Calculate the Euclidean distance $d_{i}^{+}$ to the positive ideal solution and the Euclidean distance $d_{i}^{-}$ to the negative ideal solution for each scenario.


(15) $$\begin{array}{l} d_{i}^{+}=\sqrt{\overset{n}{\underset{j=1}{\sum }}{\left(y_{ij}-y_{j}^{+}\right)}^{2}}i=1,2,\ldots ,m \end{array}$$

(16) $$\begin{array}{l} d_{i}^{-}=\sqrt{\overset{n}{\underset{j=1}{\sum }}{\left(y_{ij}-y_{j}^{-}\right)}^{2}},\;i=1,2,\ldots ,m \end{array}$$

Calculate the relative closeness *C*_*i*_ of each scheme concerning the ideal solution and rank all schemes according to the value of *H*_*i*_.


(17) $$\begin{array}{l} C_{i}=\frac{d_{i}^{-}}{d_{i}^{+}+d_{i}^{-}},\;i=1,2,\ldots ,p \end{array}$$

It can be seen that *C*_*i*_ ∈ (0, 1], a higher value of *C*_*i*_ means that the solution is closer to the optimum, and vice versa, closer to the worst. After ranking all the solutions, the solution preference and decision are made according to the final result.

## Analysis and results

**Figure 2:**
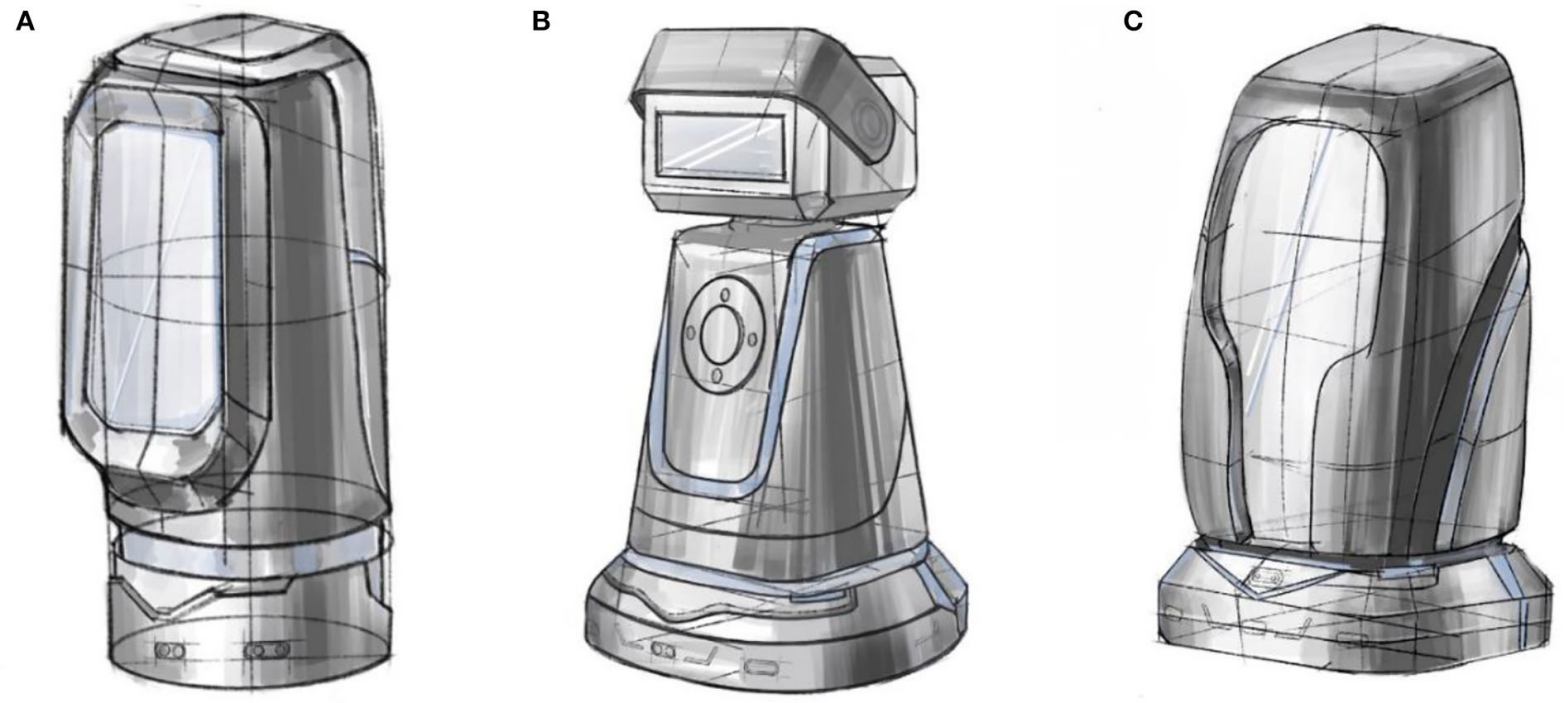
Design solutions. **(A)** Scenario a is equal to Scenario 1, **(B)** Scenario b is equal to Scenario 2, and **(C)** Scenario c is equal to Scenario 3.

The purpose of smart aging is for the use of smart products to help the elderly to age healthily with the principle of data sharing and privacy protection (41). The elderly population needs more care and companionship, and children cannot be with them all the time. Such companion smart products can enhance the happiness of the elderly, help them live more conveniently, and promote the health of their elderly life. The project team has designed a total of three solutions for intelligent escort robots for the elderly, which are selected in this paper as the evaluation object, as shown in Figure 2, and the new comprehensive evaluation method proposed in the paper was used to make a preferential ranking of them.

### Evaluation levels and indicators establishment

#### User sample selection

Considering that the elderly are a special group of people with weak information literacy and expression ability, the selection of interviewees should be extensive, and finally, 30 elderly people were selected as the interviewees of this study, including different genders (16 men and 14 women), different age groups (10 persons aged 55–60; 14 persons aged 61–65; 6 persons aged 66–70), different education levels (elementary school and below, junior high school and above, college and University, and bachelor's degree and above), and different familiarity with intelligent escort products (occasional use, more familiar, and frequent use), and the interview time was 20–30 min.

#### Primary data collection

Following the interview rules proposed in Section Theoretical overview, in-depth interviews were conducted with 30 older adults based on a semi-structured interview outline (Schedule S3 Table) (42), and they were encouraged to speak freely but not induced at appropriate times during the interview, and recorded by means of audio recording after obtaining the consent of the interviewees.

#### Open coding

The recordings were converted to text and analyzed with the aid of decoding using the Nvivo12 software. The process of open coding is to conceptualize the source material, analyzing it word by word and extracting concepts and categories in an objective manner while avoiding the influence of existing research and personal factors. Through continuous summarization, analysis, and refinement of the original information, 17 categories are finally obtained in the paper. The open decoding process is shown in Table 1.

**Table 1:** Open coding process.

**Representative statements of original information**	**Initial concept**	**Category**
The placement of buttons is not convenient for the operation	Function buttons are well placed	Interface layout rationalization
The color of the words in the interface and the background color are not very different. They will be mixed together, and cannot be seen	The use of color is more conspicuous	Color Matching
Some words are so small that you can't read them without glasses	The font of important instructions should be large	Font Matching
Some functions have too many steps and can be confusing to use	The operation steps should be simple and straightforward	Convenience of operation
I find it very convenient to be able to talk directly by voice	Wake up by direct voice or gesture operation	Interaction pattern naturalness
The human-like appearance of the escort product makes me feel unnatural	The appearance is not rigid and innovative	Novelty of appearance
More like that material is more smooth and does not look cold	Lightweight, smooth and comfortable material	Material comfort
What a function suggests doesn't make me understand what it means	Functional semantics in line with elderly cognition	Product Adaptability
The warm exterior color makes us old people at home feel comfortable and warm	Warm color tones have a sense of intimacy	Color Affinity
I hope there will be a reply soon for my operation whether it is correct or not	Give quick feedback on user operations	Immediacy
Sometimes the feedback like vibrating and flashing lights are not much guidance for my next operation	Whether the feedback is useful for user actions	Validity
Sometimes some of the feedback can be wrong	The semantic expression of the feedback is correct	Accuracy
Because the hands and feet are not very flexible, often touch by mistake, this situation will also have a reaction to the product, the elderly will be nervous	No random alarms for user misuse	Fault Tolerance
We have poor learning ability, some functions can not be taught several times to use	For each function can be learned with little effort	Easy to understand and learn
Hope more functions and cheaper	Inexpensive and functional to meet demand	Practicality
My children are not around, sometimes I would like to video with them	There are parent-child interactions and other human care functions	Caring and friendly
I hope there will not be regular problems, because we are not able to repair them	No frequent problems, more stable	Stable and durable

#### Axial coding

The spindle decoding is to further summarize and refine the mutually independent concepts and categories to arrive at the main category of the research problem. After the 18 categories obtained from the open decoding were pushed, organized, and itemized several times, five main categories were finally refined, which are human–computer interaction, product design, feedback methods, and user experience. The main axis decoding process is shown in Table 2.

**Table 2:** Axial coding process.

**Main category**	**Sub-categories**	**Connotation**
Human-computer interaction	Interface layout rationalization	Reasonable layout of operation interface (buttons, screens)
	Color Matching	The correct color match is convenient for the elderly to identify
	Font Matching	The rational use of fonts in the interface reduces the cognitive difficulty
	Convenience of operation	No complicated operation steps required
	Interaction pattern naturalness	The easiest and most convenient way to wake up and command the terminal
Product Design	Novelty of appearance	Novel appearance but without losing care for the elderly
	Material comfort	Reasonable materials to improve the comfort of use
	Product Adaptability	The semantic meaning conveyed by the interaction operation matches the cognition of the elderly
	Color Affinity	The color is not too beautiful
Feedback method	Immediacy	The feedback given can be quickly and easily understood by older adults
	Validity	The information conveyed is guiding and helpful for the elderly to operate
	Accuracy	Whether the semantics of the feedback message is consistent with the purpose of the user's operation
	Fault Tolerance	The degree to which the feedback system is fault-tolerant to malfunctions
User Experience	Easy to understand and learn	Easy to learn operation steps
	Practicality	Practical function and high-cost performance
	Caring and friendly	Humanistic care for the elderly
	Stable and durable	Less prone to breakdowns and long life

#### Selective coding

Taking the logical relationship as the starting point to explore the path relationship, structural relationship and connotation between them, the open decoding process is shown in Table 3. After selective decoding, the results need to be tested for theoretical saturation. Using the five copies of the original data reserved for the realization of the three-stage coding according to the above process, no new concepts or categories were generated, according to which theoretical saturation can be known.

**Table 3:** Selective coding process.

**Typical path relationships**	**Nature of relationship**	**Connotation**
Human-computer interaction → User satisfaction → Design decisions	Agency Relations	Whether human-computer interaction is natural affects user satisfaction and thus indirectly influences decision making
Product Design → User satisfaction → Design decisions	Agency Relations	Product design affects user satisfaction and thus indirectly influences decision making
Feedback method → User satisfaction → Design decisions	Agency Relations	Feedback methods can affect user satisfaction and thus indirectly influence decision making
User Experience → User satisfaction → Design decisions	Agency Relations	Whether the user experience is good or not affects user satisfaction and thus indirectly affects decision making

#### Theoretical modeling

**Figure 3:**
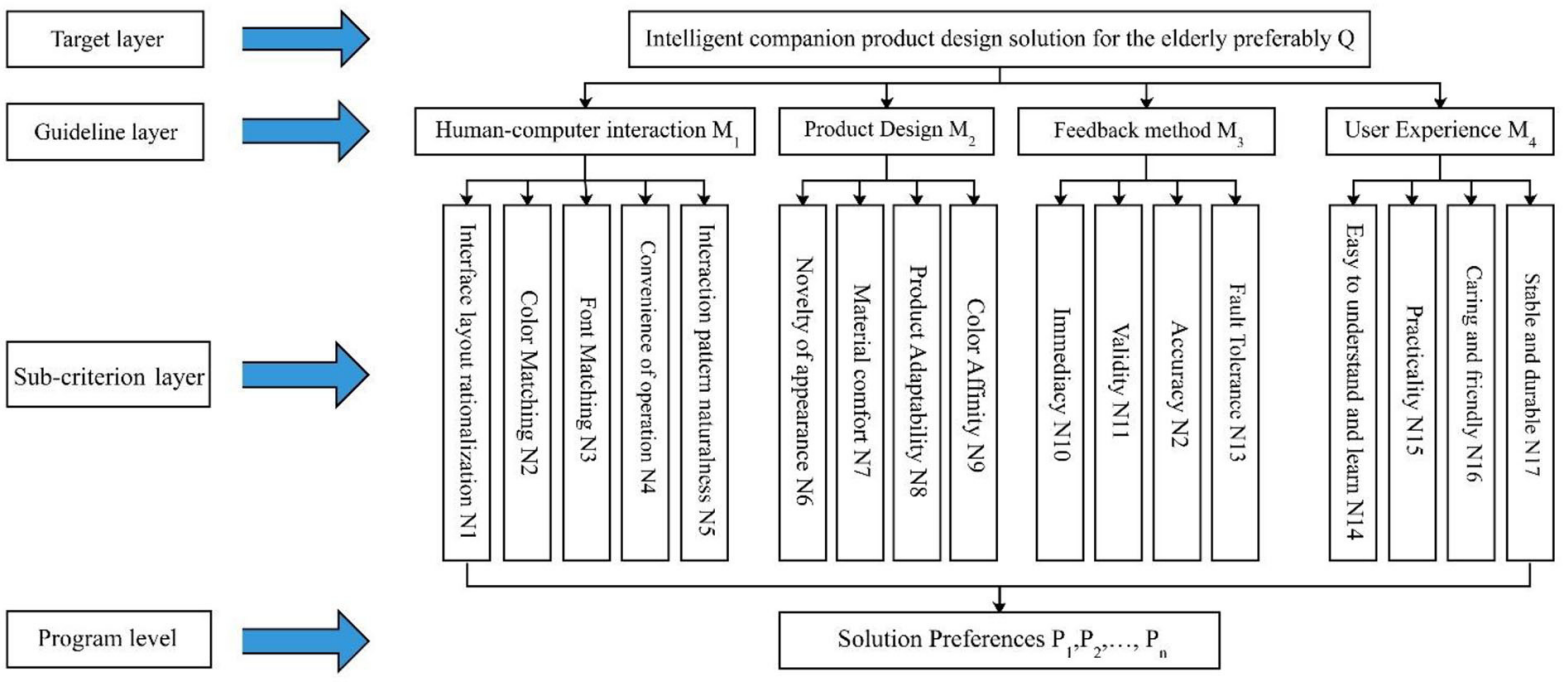
Evaluation hierarchy model of intelligent companionship products for the elderly.

Through the above research process, it can be seen that there are four factors that affect the satisfaction of elderly users with intelligent escort products, so the four main categories and 17 sub-categories can be used as evaluation indexes for design solution preference decisions and to build evaluation hierarchy. The evaluation hierarchy model of elderly smart companion products is shown in Figure 3.

### Determination of the weight of each indicator combination

#### Subjective weighting calculation

To determine the subjective weight of each evaluation criterion, five experts in the field of escort product research were selected for unified evaluation. Three of them are professors from domestic universities, specializing in elderly health care research, intelligent product design and guide design research, and have been engaged in the teaching industry for more than 20 years. Two are designer directors, engaged in the design and development of intelligent monitoring and escort products, with about 10 years of product design and development experience, and the fuzzy mutual inverse judgment matrix was obtained after the fuzzy scoring was completed using the 1–9 scale according to Equations (1) and (2), and the scores of all evaluators were summarized and collated according to Equation (3) to obtain all the comprehensive fuzzy mutual inverse judgment matrices.

According to Equation (4), the defuzzification process is carried out, and the weight values of each evaluation index in the criterion layer are calculated. Then, the consistency test is performed on each judgment matrix to obtain the final evaluation index weights and the consistency ratio of each judgment matrix, and the results are shown in Tables 4–8.

**Table 4:** Consistency ratios of the first-level criterion judgment matrix and their weights.

**A**	***M*_1_**	***M*_2_**	***M*_3_**	***M*_4_**	***W*_1_**	
*M*_1_	1.0000	3.3300	3.0000	0.3975	0.2766	λ_max_ = 4.1931
*M*_1_	0.3003	1.0000	0.2600	0.1650	0.0660	
*M*_1_	0.3333	3.8462	1.0000	0.3775	0.1634	*CR* = 0.0723
*M*_1_	2.5157	6.0606	2.6490	1.0000	0.4940	

**Table 5:** Consistency ratio of HCI criterion judgment matrix and its weights.

**M_1_**	**N_1_**	**N_2_**	**N_3_**	**N_4_**	**N_5_**	**W_2_**	
*N*_1_	1.0000	4.3300	7.0000	0.2600	0.3775	0.0465	λ_max_ = 5.4137
*N*_2_	0.2309	1.0000	4.0000	0.1725	0.2125	0.0190	
*N*_3_	0.1429	0.2500	1.0000	0.1350	0.1450	0.0087	*CR* = 0.0923
*N*_4_	3.8462	5.7971	7.4074	1.0000	3.0000	0.1293	
*N*_5_	2.6490	4.7059	6.8966	0.3333	1.0000	0.0731	

**Table 6:** Product design criteria judgment matrix consistency ratio and its weights.

**M_2_**	**N_6_**	**N_7_**	**N_8_**	**N_9_**	**W_3_**	
*N*_6_	1.0000	0.3975	0.2125	0.2725	0.0051	λ_max_ = 4.2624
*N*_7_	2.5157	1.0000	0.3775	3.0000	0.0168	
*N*_8_	4.7059	2.6490	1.0000	4.0000	0.0345	*CR* = 0.0983
*N*_9_	3.6697	0.3333	0.2500	1.0000	0.0096	

**Table 7:** Consistency ratio of judgment matrix of feedback mode criteria and its weights.

**M_2_**	**N_10_**	**N_11_**	**N_12_**	**N_13_**	**W_4_**	
*N*_10_	1.0000	0.3975	0.2725	3.6700	0.0250	λ_max_ = 4.1680
*N*_11_	2.5157	1.0000	2.0000	5.6700	0.0729	
*N*_12_	3.6697	0.5000	1.0000	5.3300	0.0558	*CR* = 0.0629
*N*_13_	0.2725	0.1764	0.1876	1.0000	0.0097	

**Table 8:** Consistency ratio of user experience criteria judgment matrix and its weights.

**M_2_**	**N_14_**	**N_15_**	**N_16_**	**N_17_**	**W_4_**	
*N*_14_	1.0000	0.3775	0.1450	3.0000	0.0501	λ_max_ = 4.2313
*N*_15_	2.6490	1.0000	0.1625	3.3300	0.0862	
*N*_16_	6.8966	6.1538	1.0000	7.3300	0.3308	*CR* = 0.0866
*N*_17_	0.3333	0.3003	0.1364	1.0000	0.0269	

#### Objective weighting calculation

**Figure 4:**
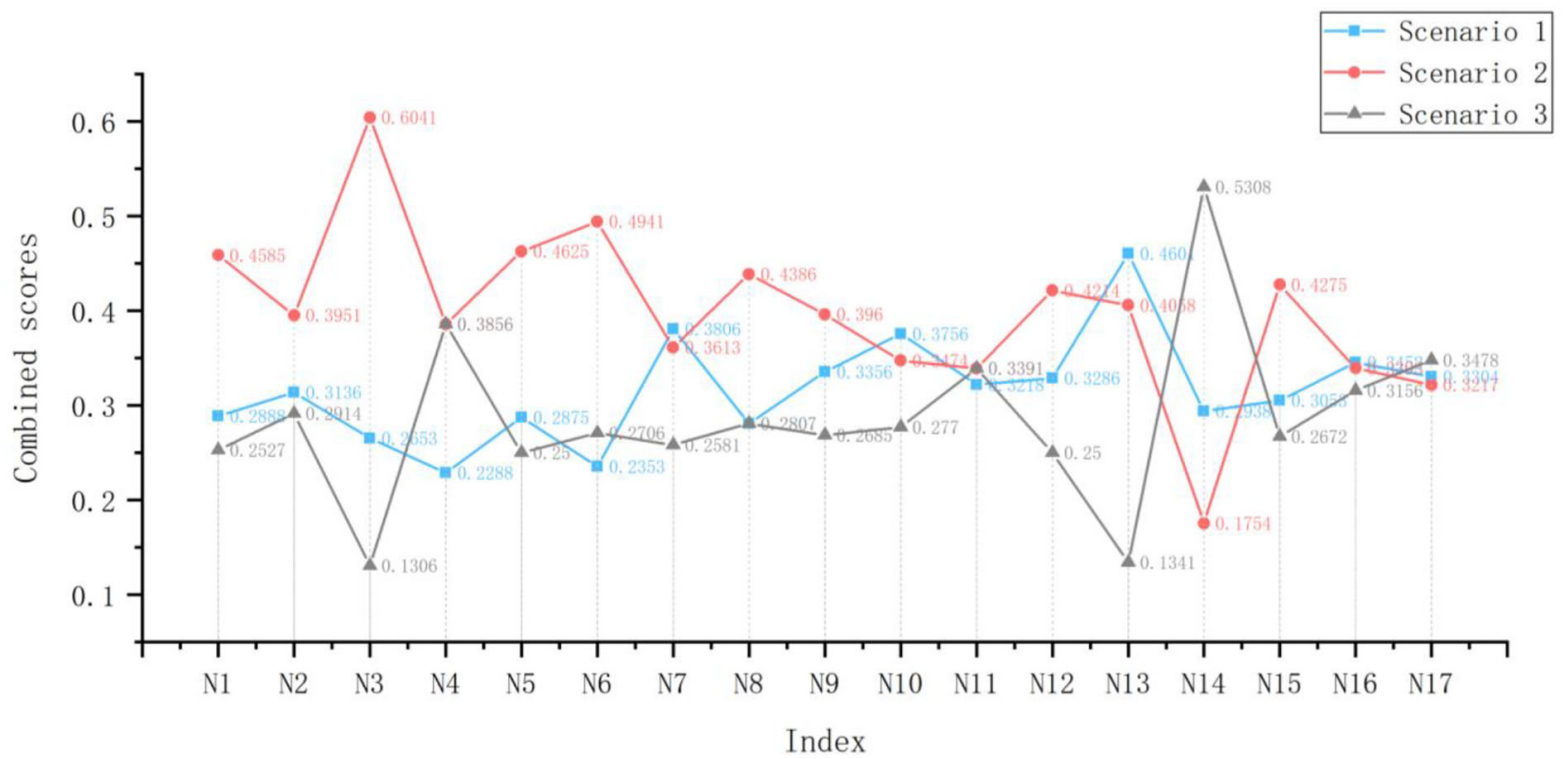
Combined scores of the programs after normalization. Scenario a is equal to Scenario 1, Scenario b is equal to Scenario 2, Scenario c is equal to Scenario 3.

The above three experts were again invited to evaluate the 17 evaluation indicators of the three programs using the fuzzy numbers of linguistic variables shown in Schedule S2 Table to construct the initial evaluation decision matrix, and the comprehensive evaluation value of each program was obtained after aggregating the evaluation values of multiple evaluators according to Equation (7). The mean area method was used to defuzzify according to Equation (8) and normalized according to Equation (9) to obtain the final composite score, which is shown in Figure 4 and Schedule S4 Table. After that, the entropy values and objective weights are found according to Equations (5) and (6), as shown in Table 9.

**Table 9:** Entropy values and objective weights of evaluation indicators.

	***H_*j*_***	***W_*j*_***
*N*_1_	0.9684	0.0540
*N*_2_	0.9920	0.0136
*N*_3_	0.8396	0.2735
*N*_4_	0.9761	0.0407
*N*_5_	0.9663	0.0575
*N*_6_	0.9489	0.0871
*N*_7_	0.9876	0.0211
*N*_8_	0.9783	0.0371
*N*_9_	0.9888	0.0191
*N*_10_	0.9928	0.0123
*N*_11_	0.9997	0.0005
*N*_12_	0.9798	0.0344
*N*_13_	0.9035	0.1646
*N*_14_	0.9115	0.1509
*N*_15_	0.9814	0.0317
*N*_16_	0.9993	0.0012
*N*_17_	0.9995	0.0008

#### Game theory portfolio weight calculation

**Figure 5:**
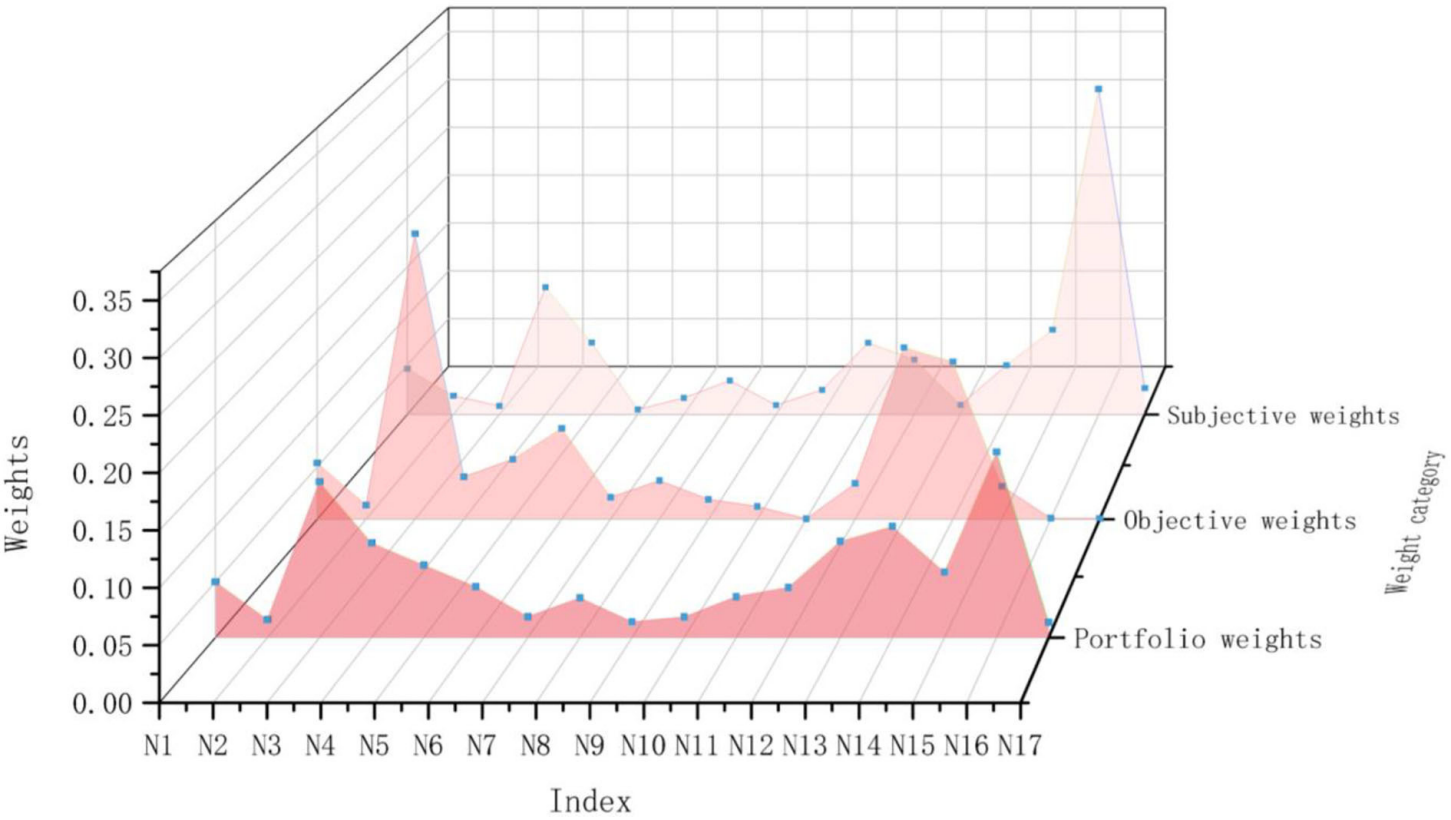
Comparison of the three weight values.

According to Equations (10)–(12), the subjective and objective weights are linearly optimized and combined to obtain the combination weights of each index, which are shown in Schedule S5 Table. The comparison of the three weights is shown in Figure 5, and it can be seen that the combined assignment of opportunity game theory clearly combines the advantages of the two assignment methods, making the final weights more stable and objective.

### Scheme sorting and selection

**Figure 6:**
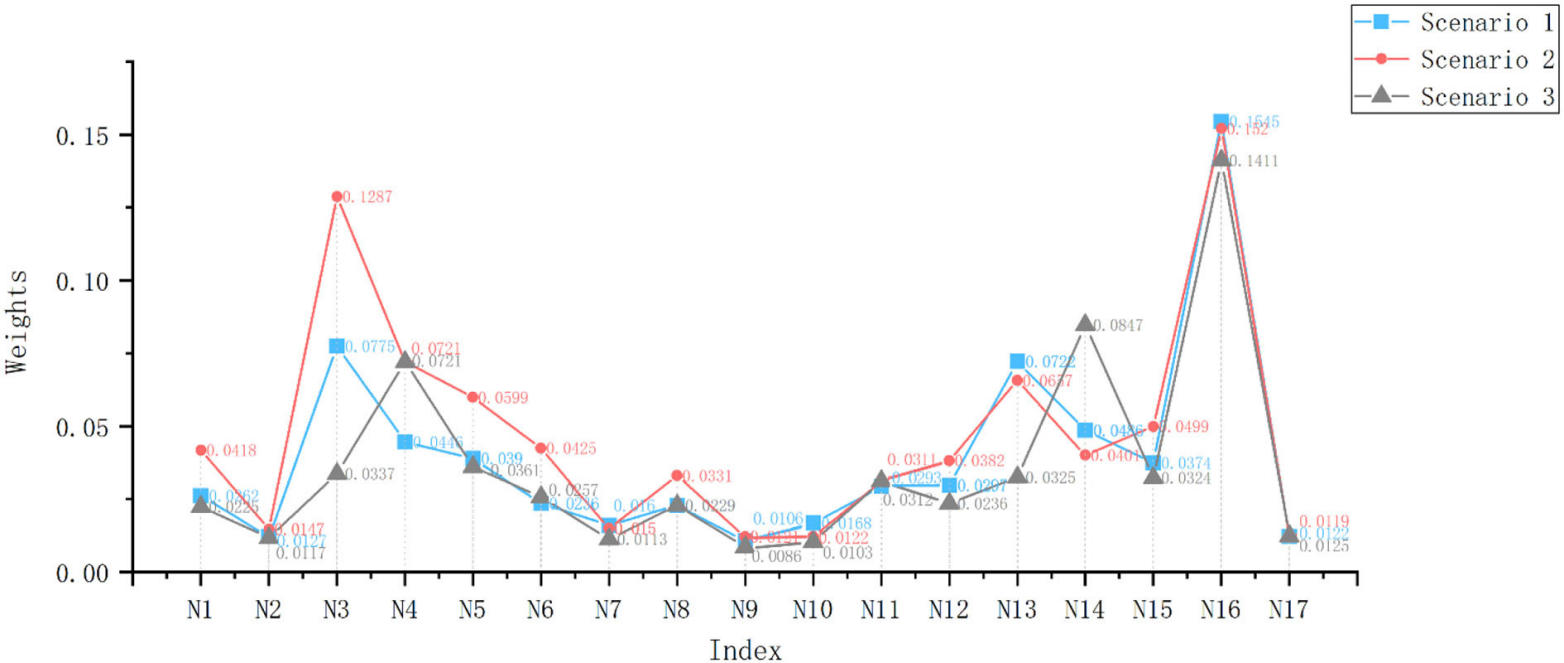
Weighted standardized decision matrix.

The initial decision evaluation constructed in the entropy weight coefficient method for objective weights is dimensionless according to Equation (13) to obtain the standardized decision matrix X. The matrix is then transformed into a weighted standardized decision matrix Y according to Equation (14), as shown in Figure 6 and Schedule S6 Table.

Finding the positive and negative ideal solutions is based on the weighted normalized decision matrix. To facilitate the operation, all elements in the weighted normalized decision matrix *Y* are expanded equally by a factor of 100, and the positive ideal solution *Y*^+^ and the negative ideal solution *Y*^−^ are found after two decimal places are retained.


$$\begin{array}{l} Y^{+}=\left\{\begin{array}{c} 4.18,1.47,12.87,7.21,5.99,4.25,1.60,3.31,\\ 1.21,1.68,3.11,3.82,7.22,8.47,4.99,15.20,1.25 \end{array}\right\}\\ Y^{-}=\left\{\begin{array}{c} 2.25,1.17,3.37,4.46,3.61,2.36,1.13,2.29,\\ 0.86,1.03,2.93,2.36,3.25,4.01,3.24,14.11,1.19 \end{array}\right\} \end{array}$$

The Euclidean distances $d_{i}^{+}$ and $d_{i}^{-}$ and the relative closeness *C*_*i*_ of each scheme to the positive and negative ideal solutions are calculated according to Equations (15)–(17), and the ranking is completed according to the size of *C*_*i*_, as shown in Table 10.

**Table 10:** Euclidean distance and relative closeness.

**Scenario**	** ${\text{d}}_{\text{i}}^{+}$ **	** ${\text{d}}_{\text{i}}^{-}$ **	**C_i_**
*a*	7.7906	6.244389	0.444915
*b*	14.41117	16.9398	0.540328
*c*	11.24728	5.247657	0.318138

By judging the value of *C*_*i*_, the larger the value of *C*_*i*_, the closer the scheme is to the positive ideal solution, and the farther it is from the negative ideal solution, the better the scheme is. It can be found through Table 10 that scenario b is obviously better than the other two schemes.

### Feasibility assessment

**Figure 7:**
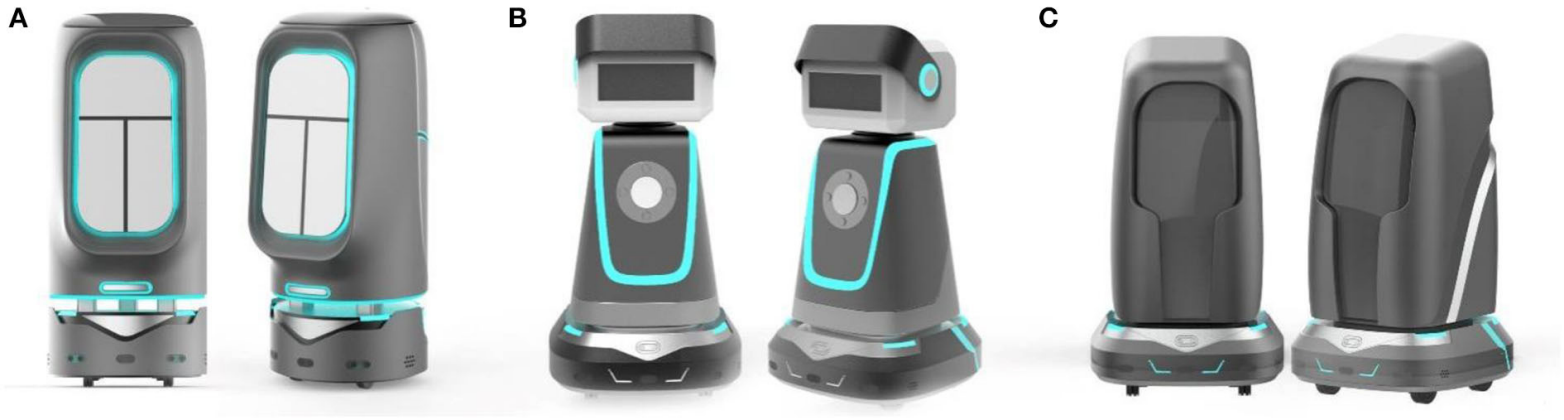
Functional prototypes. **(A)** Scenario a is equal to Scenario 1, **(B)** Scenario b is equal to Scenario 2, and **(C)** Scenario c is equal to Scenario 3.

To verify whether the evaluation results are correct and reasonable and whether the evaluation methods are practical and feasible, researchers often use some standardized questionnaires to measure user's experience, so as to verify the reasonableness of the evaluation results. Mainstream standardized questionnaires used internationally are Questionnarie for User Interaction Satisfaction(QUIS) (43), The Post-Study System Usability Questionnaire (PSSUQ) (44), The System Usability Scale (SUS) (45), etc. The feasibility test using a standardized questionnaire not only allows us to judge the correctness of the evaluation results but also allows us to understand through user feedback whether the preferred solution is effective in terms of the degree of health of the elderly in their old age. The above three solutions were made into functional prototypes, as shown in Figure 7, and 100 elderly people with experience in using intelligent escort products in a senior citizen University in Wuhan were selected for experimental verification. The 100 participants included different genders (54 males and 46 females), different age groups (51 persons aged 55–65; 42 persons aged 66–75; 7 persons aged 76 and above), and different educational levels (26 persons with compulsory education level), 32 people with high school education level, 42 people with college education level or above), different intelligent product service years (3 people under 1 year, 38 people in 2–5 years, 59 people over 5 years). A total of 100 elderly users were asked to use each of the three functional prototypes and fill out the PSSUQ questionnaire based on their experience after use. The questionnaire was used to compare the three home recreation and care companion robots and verify the evaluation results through user satisfaction with the three products.

The PSSUQ is used to assess the user's perceived satisfaction with a computer system, product, or application and is consistent with the goal of assessing user satisfaction with an escort robot user. Questionnaire style reference is shown in Schedule S7 Table. The questionnaire was scored on a 7-point Likert scale, with 1 for strongly disagree and 7 for strongly agree. The overall assessment usability questionnaire has four indicators: Overall, System quality, Information quality, and Interface quality. Each indicator is averaged over the set of its corresponding question items, with higher scores indicating higher satisfaction and vice versa.

**Figure 8:**
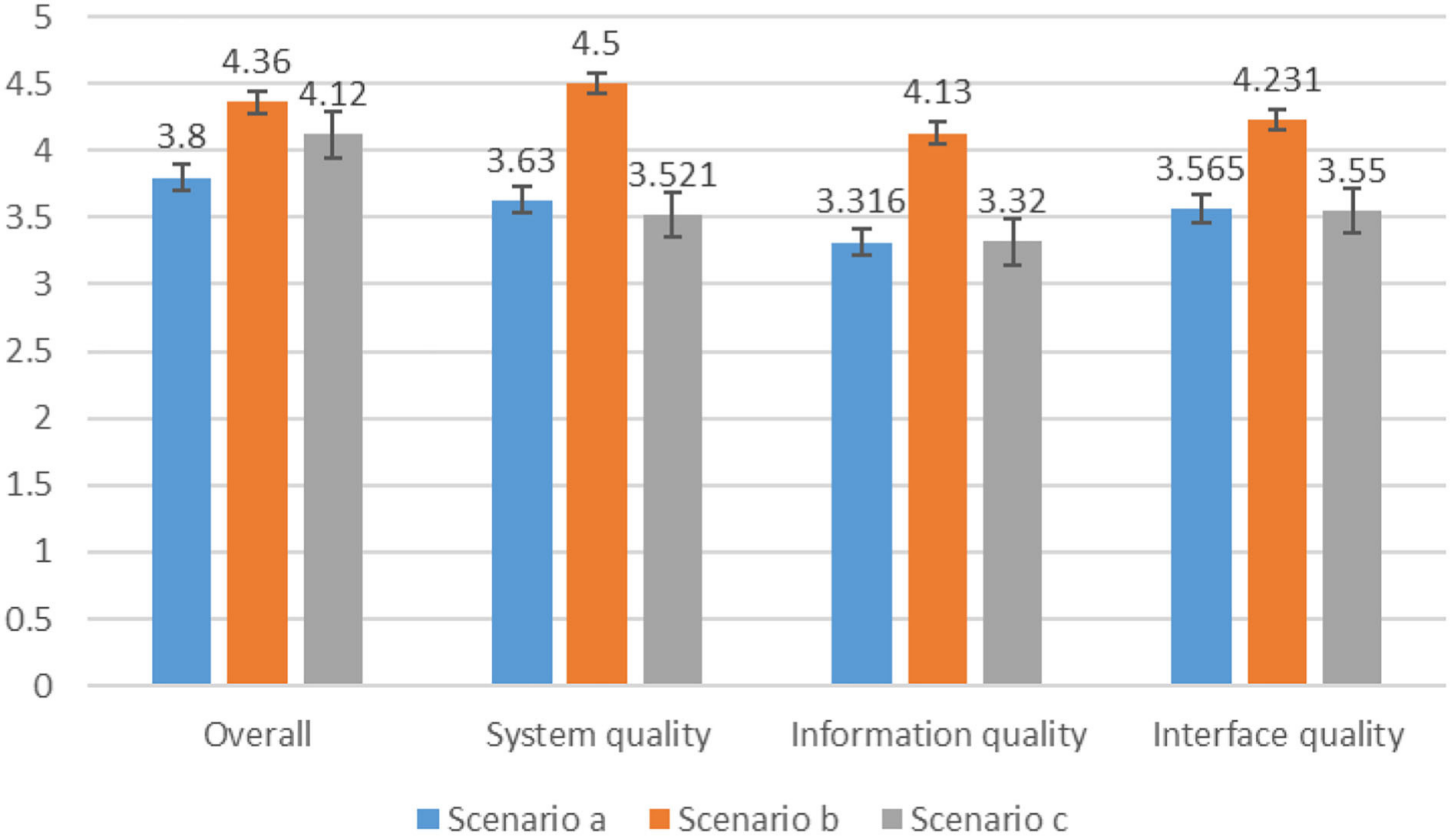
PSSUQ evaluation scores for the three scenarios.

A total of 100 questionnaires were distributed, and 94 valid questionnaires were finally obtained through screening. The alpha coefficient of the questionnaire was calculated by SPSS 26.0 as 0.991, indicating that the reliability of the questionnaire was high and met the requirements. The four indicators of overall, system quality, information quality, and interface quality of the three solutions were compared and analyzed. Figure 8 shows that scenario b scored significantly higher than the other two solutions, and the results are consistent with the results obtained by using the game theory combination of weighting and TOPSIS method, indicating that the above method is feasible to be applied to the evaluation of recreation and health intelligent product design. At the same time, according to the feedback of 100 elderly users, all said that scenario b is more suitable for use than the other two scenarios, which can bring more convenience in life and promote mental and life health.

## Discussion

Regarding research on older adults, many scholars currently focus on physical health studies of older adults or those factors that can have an impact on older adults' cognition, such as Noguchi et al. (46) who used multiple linear regression methods to study the effects of social relationships on older adults' cognitive abilities. Few studies have been conducted on the evaluation of intelligent escort products used by older people. The development of a comprehensive evaluation method will not only help designers to evaluate design solutions more objectively and rationally, but also preferably select the product solution that best meets the needs of older people and ultimately benefits them.

First, the article coded a large amount of user interview data at three levels based on the grounded theory and constructed theoretical models as evaluation indexes scientifically and rationally by qualitative research methods, which to some extent overcame the defects of many scholars in constructing evaluation systems based on their own experience and subjective induction of evaluation indexes. Second, the article uses game theory combination assignment as the quantitative analysis method to calculate the final weights of each evaluation index, makes the preferential decision of the scheme according to the TOPSIS method, and also introduces triangular fuzzy numbers to transform the fuzziness in the evaluation process of qualitative indexes into the corresponding fuzzy numbers for quantification, which reduces the evaluation inaccuracy caused by the fuzziness of human thinking in the evaluation process.

In the introduction part of the article, we also put forward the relationship between health care and product design solutions. With a mature solution decision-making theory or method, designers can well avoid the blindness of choice, so that high-quality products can flow into the market to enhance the experience of the elderly and promote healthy elderly life. In the decision-making process, for multi-criteria decision-making, the single use of the scoring method will cause inconsistencies between the evaluation results. The comprehensive score of the indicators in Figure 4 is the calculation result of the single use of the entropy weight method. The indicators *N*_3_, *N*_6_, *N*_13_, *and N*_14_, have a large gap between the scores of different schemes. However, after using the comprehensive evaluation method, the idea of minimizing dispersion will complement the advantages of the subjective and objective weights. It is not difficult to see from the comparison of the three weights in Figure 5 that the comprehensive weight is more stable than the other two weights, and there will be no maxima or minima. In addition, Figure 6 shows us that the index weight difference of different schemes is within a small range of change. When we map the index evaluation to the scheme optimization decision, it is difficult to directly observe the pros and cons of the scheme, while TOPSIS provides us with an opportunity to achieve decision-making through rational and scientific calculations.

## Strengths and limitations

The new comprehensive evaluation method proposed in the article is better than the traditional evaluation method, solves the problem of multiplier effect in the determination of weight values in the general evaluation method, is more scientific and objective for the determination of evaluation indexes, saves the production cost of enterprises, and indirectly improves the quality of healthy life of the elderly.

There are some limitations in the study regarding self-reported data. First, as a special group, older people have limited cognitive abilities and have an inherent mindset about smart products, which can lead to overstated and unrealistic interview results. Second, the population interviewed was not divided in detail; the cognitive abilities of older people in rural areas are different from those in urban areas, and the results of the interviews will differ between people in different areas. Motohiro et al. (47) investigated the role of community environmental factors in cognitive performance in old age. Therefore, this study could be explored in the future for different age groups or different regions of the elderly population to analyze whether differences in the environment affect the judging results.

The measures used to collect data in this study remain implausible. The scoring process in the study was subjective, and although the use of fuzzy thinking avoided subjectivity to a certain extent, the number of experts scoring was small, and the results obtained were not completely objective and had limitations. A follow-up study will use the instrument to collect relatively objective data as an evaluation set, which will lead to further refinement of the evaluation.

The focus of this stage of research is to propose an evaluation method to help designers make product decisions and to show the possibility of comprehensive evaluation in the optimization of health care products. Aiming at the problem that the overall process of the proposed evaluation method is complicated and the cost is high in the daily use of designers, the focus of the next stage of this research is to compare the analysis results of different multi-attribute decision-making methods, select the optimal solution, improve and simplify the comprehensive evaluation method, and enhance the practicality of the method.

## Conclusion

Due to the decline in cognitive ability, mobility, and information literacy of the elderly, they are increasingly disconnected from information technology products and have barriers to the use of smart products in their lives, which seriously reduces the healthiness of senior living. Designers often generate multiple design solutions based on the multiple needs of older adults, and it is critical to use reasonable evaluation methods to decide on the optimal solution. In this paper, a new comprehensive evaluation method is proposed using a combination of qualitative research methods and statistical methods, three escort robot solutions are preferentially evaluated, and reasonable results are finally obtained. The article concludes with an assessment of the reasonableness of the evaluation results and the feasibility of the method, which shows that the method is highly feasible and helpful for designers to conduct program evaluation and improve the health of elderly people's retirement life.

## Data availability statement

The original contributions presented in the study are included in the article/Supplementary material, further inquiries can be directed to the corresponding author/s.

## Ethics statement

The studies involving human participants were reviewed and approved by the Ethics Committee of Hubei University of Technology. The patients/participants provided their written informed consent to participate in this study.

## Author contributions

SH and QJ are responsible for the identification and review of core elements such as research ideas and experimental protocols. MG is responsible for the writing of the manuscript. LD and JH were responsible for the compilation of the interview data and the drawing of the figures. WG was responsible for the final corrections to the manuscript. All authors listed have made a substantial, direct, and intellectual contribution to the work and approved it for publication.

## Funding

This study was supported by the National Social Science Foundation, Fund Number: 15CG147, the Second Batch of Hubei Stage Art Talents and Art Talents Training Project, Fund Number: EWTO [2018] No. 5, and Major Project of Hubei Provincial Philosophy and Social Science Research, Fund number: 21ZD053. The funding body was not involved in the design of the study, data collection, analysis, interpretation, and as well as in writing the manuscript.

## Conflict of interest

The authors declare that the research was conducted in the absence of any commercial or financial relationships that could be construed as a potential conflict of interest.

## Publisher's note

All claims expressed in this article are solely those of the authors and do not necessarily represent those of their affiliated organizations, or those of the publisher, the editors and the reviewers. Any product that may be evaluated in this article, or claim that may be made by its manufacturer, is not guaranteed or endorsed by the publisher.
