# Supplementary material for: An evaluation method for product design solutions for healthy aging companionship
Source: Front Public Health. 2022 Sep 20;10:919300. doi: 10.3389/fpubh.2022.919300 (PMC9530995; doi:10.3389/fpubh.2022.919300)
Supplement: Supplementary file 1 [file Table_1.pdf]

Supplementary information（Schedule）

S1 Table. 1-9 Quantification and meaning of the linguistic terms of the scaling scale

Scale	Judgment	The relative importance between elements i and j	Fuzzy number	Countdown
1	Equally important	Comparison of two indicators is equally important	1,1,1	1,1,1
2			1,2,3	1/3,1/2,1
3	Slightly more important	One metric is slightly more important than the other	2,3,4	1/4,1/3,1/2
4			3,4,5	1/5,1/4,1/3
5	Obviously important	One metric is significantly more important than the other	4,5,6	1/6,1/5,1/4
6			5,6,7	1/7,1/6,1/5
7	Very important	One metric is very important over another	6,7,8	1/8,1/7,1/6
8			7,8,9	1/9,1/8,1/7
9	Extremely important	The extreme importance of one metric over another	8,9,10	1/10,1/9,1/8

(Note: 2,4,6,8 are the compromise values, r_{ij}=${r_{ij}}^{-1}$. One indicator is less important than the other, using the reciprocal representation)

S2 Table. Correspondence between linguistic terms and triangular fuzzy numbers

Language items	Triangular fuzzy number
Very poor（VVP）	（0,0,0）
(VP)	（0,0,0.2）
Poor (P)	（0,0.1,0.3）
(MP)	（0.1,0.2,0.4）
Moderate (F)	（0.3,0.5,0.7）
(MG)	（0.6,0.7,0.8）
Good (G)	（0.7,0.8,0.9）
(VG)	（0.8,0.9,1）
Very good (VVG)	（0.9,1,1）

S3 Table. Semi-structured interview outline

Serial number	Possible questions to ask the interviewee
1	How much do you need escort products in your life? Do you use escort products regularly?
2	What is your opinion on the interface design of smart escort products?
3	What kind of interaction do you expect from an intelligent escort product?
4	What do you expect from the appearance, material and color of the products?
5	What kind of feedback do you expect on your actions?
6	Can you understand the guidelines for the operation of the escort product?
7	What other features do you think smart escort products need to have?

S4 Table. Combined scores of the programs after normalization

	Scenario 1	Scenario 2	Scenario 3
N_1	0.2888	0.4585	0.2527
N_2	0.3136	0.3951	0.2914
N_3	0.2653	0.6041	0.1306
N_4	0.2288	0.3856	0.3856
N_5	0.2875	0.4625	0.2500
N_6	0.2353	0.4941	0.2706
N_7	0.3806	0.3613	0.2581
N_8	0.2807	0.4386	0.2807

N_9	0.3356	0.3960	0.2685
N_{10}	0.3756	0.3474	0.2770
N_{11}	0.3218	0.3391	0.3391
N_{12}	0.3286	0.4214	0.2500
N_{13}	0.4601	0.4058	0.1341
N_{14}	0.2938	0.1754	0.5308
N_{15}	0.3053	0.4275	0.2672
N_{16}	0.3452	0.3393	0.3156
N_{17}	0.3304	0.3217	0.3478

S5 Table. Weight of each indicator combination

	Subjective weights	Objective weights	Portfolio weights
N_1	0.0465	0.0540	0.0502
N_2	0.0190	0.0136	0.0163
N_3	0.0087	0.2735	0.1403
N_4	0.1293	0.0407	0.0853
N_5	0.0731	0.0575	0.0653
N_6	0.0051	0.0871	0.0459
N_7	0.0168	0.0211	0.0189
N_8	0.0345	0.0371	0.0358
N_9	0.0096	0.0191	0.0143
N_{10}	0.0250	0.0123	0.0187
N_{11}	0.0729	0.0005	0.0369
N_{12}	0.0558	0.0344	0.0452
N_{13}	0.0097	0.1646	0.0867
N_{14}	0.0501	0.1509	0.1002
N_{15}	0.0862	0.0317	0.0591
N_{16}	0.3308	0.0012	0.1670
N_{17}	0.0269	0.0008	0.0139

S6 Table. Weighted standardized decision matrix

	Scenario 1	Scenario 2	Scenario 3
N_1	0.0262	0.0418	0.0225
N_2	0.0121	0.0147	0.0117
N_3	0.0775	0.1287	0.0337
N_4	0.0446	0.0721	0.0721
N_5	0.0390	0.0599	0.0361
N_6	0.0236	0.0425	0.0257
N_7	0.0160	0.0150	0.0113
N_8	0.0229	0.0331	0.0229
N_9	0.0106	0.0121	0.0086
N_{10}	0.0168	0.0122	0.0103
N_{11}	0.0293	0.0311	0.0312
N_{12}	0.0297	0.0382	0.0236
N_{13}	0.0722	0.0657	0.0325
N_{14}	0.0486	0.0401	0.0847
N_{15}	0.0374	0.0499	0.0324

N_{16}	0.1545	0.1520	0.1411
N_{17}	0.0122	0.0119	0.0125

S7 Table. Post-Study System Usability Questionnaire

		Strongly disagree →Strongly agree									
Serial number	Questionnaire questions		1	2	3	4	5	6	7		Not applicable
1	Overall, I'm satisfied with how easy the system is to use										
2	Using this system is easy										
3	I can complete my tasks quickly with this system										
4	I feel very comfortable using this system										
5	Learning this system is easy										
6	I believe that using this system will increase output										
7	This system gives me an error message that clearly tells me how to solve the problem										
8	I can easily and quickly recover when something goes wrong with this system										
9	The information provided by this system is clear										
10	It's easy to find the information I need										
11	Information can effectively help me accomplish my tasks										
12	The information in the system screen is clearly organized										
13	The interface of this system is very comfortable										
14	I like the interface of using this system										
15	This system has all the features I expected it to have										
16	Overall, I am satisfied with the system										
*The interface includes the parts used to interact with the system, such as the microphone, the screens (including their images and text), etc.											
